# Editorial: Dementia, Frailty and Aging

**DOI:** 10.3389/fmed.2018.00168

**Published:** 2018-05-29

**Authors:** Wee-Shiong Lim, Marco Canevelli, Matteo Cesari

**Affiliations:** ^1^Department of Geriatric Medicine, Institute of Geriatrics and Active Ageing, Tan Tock Seng Hospital, Singapore, Singapore; ^2^Department of Human Neuroscience, Sapienza University of Rome, Rome, Italy; ^3^UOSD Geriatria, Fondazione IRCCS Ca' Granda - Ospedale Maggiore Policlinico, Milan, Italy; ^4^Dipartimento di Scienze Cliniche e di Comunità, Università di Milano, Milan, Italy

**Keywords:** dementia, frailty, aging, population aging, big data

Population aging is both a worldwide success story and a worldwide health conundrum, with the increasing age of populations around the world leading to unprecedented challenges (1). According to the United Nations report on World Population Prospects (2017), there is an estimated 962 million people aged 60 years and above who comprise 13% of the global population (2). The beginning of the twenty-first century has seen health systems worldwide struggling to deliver quality healthcare amidst challenges posed by aging populations (3). Traditional medicine and models of care have been premised on the evaluation and treatment of standalone and usually acute diseases occurring in relatively younger individuals. This contrasts with the current reality of multiple, interacting, and often chronic conditions affecting older persons. It is thus necessary to disentangle the pathophysiological mechanisms, clinical manifestations, and inter-relationships of age-related conditions in order to personalize clinical interventions and realign health systems to better address the unmet needs of frail older persons (4, 5).

Against this backdrop, frailty and dementia have emerged as priority areas in both research and clinical settings due to their high prevalence, impact on the individual's quality of life, and public health impact (6–8). These conditions aptly reflect the complexity of age-related pathological conditions, causally underpinned by a myriad of heterogeneous, interacting, and often unclear pathophysiological processes. Indeed, a hallmark of both conditions is the inherent difficulty in differentiating the effects of the normal aging process from the eventual pathophysiological deviations of the underlying disease (9, 10). Their occurrence and trajectories over time are strongly affected by a wide array of factors and determinants that are not confined to single biological systems and/or health domains (10). Moreover, environment and social factors also substantially influence the definition of different phenotypes. This raises the clarion call for a broader, integrated, and holistic approach that is able to more adequately capture the biological, clinical, and psychosocial complexities of frailty and dementia, thus paving the way for improvement in the consequent outcomes (11–13).

The present Research Topic represents a timely addition to the burgeoning body of evidence which aims to provide fresh perspectives in our understanding of the frailty and dementia phenomena occurring with aging. An area of particular interest is the emerging construct of cognitive frailty (CF), which is designed to operatively capture the co-existence of frailty and cognitive impairment in the absence of dementia (14). Using a modified version of the IANA/IAGG criteria (15), Ma et al. reported a 2.7% prevalence of CF in a Chinese older population. Older persons, women, and people living in rural areas were found to be at higher risk of CF. Corroborating the recommendations of the Lancet Commission report (7), depression and hearing impairment were independently associated with CF in elderly individuals with physical frailty. The study by Nyunt et al. explored the physical frailty phenotype in mild cognitive impairment (MCI). When compared with participants with “normal high cognition,” there was a higher prevalence of frailty and pre-frailty attributable to low lean mass, slow gait speed, or balance and gait impairment. In their 5-year observational study of 91 subjects with amnestic MCI, Trebbastoni et al. also noted that frailty was associated with increased risk of conversion to Alzheimer's dementia, even in those with high baseline level of cognitive performance.

Four papers in this Research Topic shed further insights to illuminate the knowledge gap in our understanding of the interface between cognitive and physical domains. In their study of 269 elderly individuals with subjective memory complaints, Hooper et al. did not find any significant cross-sectional associations between fatigue and Aβ load. However, sensitivity analysis revealed a weak association with increased Aβ in the hippocampus in subjects with MCI, thereby providing indirect support for the construct of CF at the early stages of Alzheimer's disease. Chhetri et al. proposed the motoric cognitive risk (MCR) syndrome, characterized by the simultaneous presence of gait disturbances and memory complaints in older adults, as a means to examine the close interactions between cognitive and physical domains and identify individuals at risk of dementia and other age-related adverse outcomes. By summarizing the existing evidence from both human and animal models, Bellelli et al. highlighted the multiple common pathophysiologic mechanisms and pathways of delirium and frailty, to lay out the case for delirium as the cognitive harbinger of a state of frailty in the context of an acute clinical event. This opens the door for further studies to examine the contribution of physical frailty to adverse outcomes in delirium, and conversely, the deleterious impact of delirium on physical frailty (16). Using the examples of frailty and MCI, the review by Canevelli et al. challenged the widely-held assumptions of these entities as unequivocally prodromal stages of a future disease state by providing a timely reminder of our incomplete understanding of the transitions of clinical at-risk conditions and their potential for clinical improvement and spontaneous reversion.

Dementia is a devastating and debilitating illness that has far-reaching public health, social and economic ramifications. Therefore, the Research Topic submissions also covered pertinent areas in dementia such as caregiving and diagnosis. To keep pace with the projected exponential rise in dementia, it is imperative that we tap upon the “invisible workforce” of family caregivers and understand the factors that predispose to caregiver burden (17). Li et al. confirmed the existence of the unique “worry about performance” (WaP) burden in the multidimensionality of caregiver burden beyond role and personal strain. Unlike other factors, WaP was significantly reported even in early cognitive impairment, suggesting its potential as a possible target for interventions aimed at improving self-efficacy among caregivers in the milder stages of burden (18). In their study examining the rapidly expanding group of caregivers of dementia in oldest-old (CDOO), Win et al. reported these were mainly older adult children who experienced significant role and personal strain rather than WaP while caring for their oldest-old family members with more impaired cognitive and physical function. To address the challenges of under-detection of dementia in the primary care setting, Teixeira et al. described a potentially scalable multi-stage strategy for community detection that involved initial screening by health professionals to identify at-risk individuals for more comprehensive evaluation. With the recent release of the NIA-AA Research Framework directed toward a biological definition of Alzheimer's disease (19), the real-world study of geriatrics outpatients by Dolci et al. highlighted the discrepancy between clinical diagnosis of Alzheimer's disease with cerebrospinal fluid and neuroimaging biomarkers, thereby reiterating the caution against premature and inappropriate usage of biomarker-based research frameworks in general medical practice.

Novel approaches are also suggested in this Research Topic. Reviving a 100-year old idea about a possible role played by gut microbiota in modulating brain morphology and function across the life-course (20), Calvani et al. proposed the fascinating concept of the “second brain aging” which links age-related changes in the gut mircrobiota to neurodegeneration and related conditions (including depression, Alzheimer's disease, and Parkinson's disease). This raises the tantalizing prospect of developing interventions that target the gut microbiota as part of a comprehensive strategy in dementia prevention and treatment. Yatawara et al. explicated the cognitive-anatomical basis of getting lost behavior in patients with mild Alzheimer's disease. They reported that the top-down modulation deficit is localized to the medial temporal lobe and did not follow the typical mechanism in healthy aging, highlighting the need to target both working memory and visuospatial deficits simultaneously. Lastly, the thoughtful review by Lenca et al. explored the potential of harnessing big data approaches to improve current preventive and predictive models in dementia care and research (e.g., enabling earlier diagnosis, optimizing resource allocation, and delivering individualized treatments tailored to patients). The authors highlighted technical, scientific, ethical, and regulatory challenges and proposed the need for multi-level integrative approaches to chart the route ahead for research, ethics, and policy.

As guest editors for this research topic on frailty, dementia, and aging, we are delighted to commend to you the collection of 14 articles as an important contribution to “evidence-balanced medicine” in the real world of frail older persons (21).

## Author contributions

W-SL, MarC, and MatC conceived the manuscript. W-SL drafted the paper. MatC critically appraised and edited the manuscript. All authors read and approved the final version of the paper.

### Conflict of interest statement

The authors declare that the research was conducted in the absence of any commercial or financial relationships that could be construed as a potential conflict of interest.
